# Negative Feedback Regulation of T Cells via Interleukin-2 and FOXP3 Reciprocity

**DOI:** 10.1371/journal.pone.0001581

**Published:** 2008-02-13

**Authors:** Zoran Popmihajlov, Kendall A. Smith

**Affiliations:** Division of Immunology, Department of Medicine, Weill Medical College, Cornell University, New York, New York, United States of America; New York University School of Medicine, United States of America

## Abstract

As interleukin-2 (IL2) is central to the clonal expansion of antigen-selected T cells, we investigated the relationship between IL2 and the negative regulatory transcription factor FOXP3. We found IL2 to be responsible for T cell antigen receptor (TCR)-activated FOXP3 expression by both CD4+ and CD8+ human T cells, and as anticipated, FOXP3 expression restricted TCR-stimulated IL2 expression. However, no evidence could be found that FOXP3+ cells actively suppress IL2 expression by FOXP3- cells. These data are consistent with an IL2/FOXP3-dependent negative feedback loop that normally regulates the T cell immune response. It follows that a defect in this negative feedback loop as a result of a deficiency of either IL2 or FOXP3 will lead to a hyperproliferative autoimmune syndrome, without the necessity of invoking an active suppressive function for FOXP3+ T cells.

## Introduction

Thirty years ago, when the T cell growth factor interleukin-2 (IL2) was first quantified, we found it to be produced only transiently after T cell activation by mitogens or antigens [1]. As an explanation, we postulated that perhaps there was a natural feedback inhibition operative that shut down IL2 production, thereby limiting IL2-mediated T cell proliferation during an immune response. A search for the release of a soluble inhibitor proved negative, but additional experiments revealed that IL2-responsive T cells actually consume IL2, thus providing at least one explanation for the disappearance of IL2 as the cells proliferate to high densities [1], [2], [3].

Subsequently, targeted disruption of the IL2 gene resulted in IL2 (−/−) mice with apparently normal lymphocyte development, but deficient *in vitro* T cell proliferative responses [4] . However, as these mice aged they developed a paradoxical autoimmune lymphoproliferative syndrome, with the accumulation of activated T cells in multiple organs, including salivary glands, lungs, kidneys, heart, pancreas and liver [5]. As well, autoimmune hemolytic anemia and inflammatory bowel disease ultimately led to premature death [6]. These findings led us to the hypothesis that an unanticipated crucial defect resulting from the elimination of IL2 might be a lack of a negative regulatory feedback function [7].

Our experiments to try to understand this phenomenon demonstrated that IL2 administration to IL2 (−/−) mice prevented the onset of the autoimmune syndrome. Furthermore, adoptive transfer of splenocytes and thymocytes from IL2-treated IL2 (−/−) mice delayed the onset of disease, thereby leading us to the conclusion that IL2 induces some T cell maturation/differentiation event that subsequently prevents the cells from responding to self antigens [8]. However, the precise nature of this IL2-induced cellular change remained obscure.

At about this time the *scurfy* mutant mouse [9] was found to be suffering from a similar lymphoproliferative phenotype [10], [11], which was attributed to an over-expression of cytokine genes by CD4+ T cells [12], [13], [14]. Subsequently, it was shown that *scurfy* T cells are hyper-responsive to T cell antigen receptor (TCR) triggering, which prompted the speculation that perhaps the *scurfy* phenotype results from a defect of a normal feedback down-regulation of TCR activation of cytokine gene expression [15].

A possible explanation for these observations was introduced when it was reported that CD4+ T cells expressing the α-chain of the IL2 receptor (IL2Rα) (CD25) prevent a quite similar lethal lymphoproliferative syndrome when transferred to lymphopenic (*nu/nu)* [16], and neonatal thymectomized mice [17], [18]. Additional reports confirmed these initial findings, and CD4+CD25+ T cells were proposed to represent a unique lineage of immunoregulatory T cells or “Regulatory T cells” (T-Regs) that function normally to actively suppress immune responses to potential autoantigens [19].

Subsequently, two functional characteristics of CD4+CD25+ T-Regs were described; anergy, defined by an incapacity to produce IL2 and proliferate when activated via the TCR, and as well, the capacity to actively suppress polyclonal T cell proliferation *in vitro*, via inhibition of IL2 production by TCR-activated “effector” cells [20]. It is noteworthy that high ratios of T-Reg to T-effector cells (i.e. 2∶1, 1∶1, 1∶2, 1∶4) are usually necessary to demonstrate the *in vitro* suppressive effect. Problematic with this sort of assay is the capacity for IL2R+ cells to passively bind, remove and degrade IL2, which then appears as if there is a suppressive activity, since proliferation is driven by IL2 [1], [2], [3]. Moreover, problematic in the designation of CD4+CD25+ T cells as T-Regs was that the same phenotype is shared by activated, nonanergic and nonsuppressive “effector” T cells [21].

When the *scurfy* gene was cloned [22], it was found to encode a new member of the forkhead family of transcription factors, FOXP3. When tested for activity, over expression of FOXP3 in CD4+ T cells was found to attenuate TCR-induced IL2 production and subsequent proliferation, thereby providing molecular support for the negative feedback hypothesis [23]. Moreover, coincident with the identification of the *scurfy* gene as encoding FOXP3 in mice, the human ortholog was cloned and found to be mutated in individuals suffering from the X-linked autoimmunity-allergic dysregulation (XLAAD) syndrome [24], and the X-linked neonatal diabetes mellitus, enteropathy and endocrinopathy (IPEX) syndrome [25], [26].

Soon thereafter, three almost simultaneous reports [27], [28], [29] linked mutations of FOXP3 with the lack of development of T-Regs in the mouse, thereby providing a potential explanation for the severe autoimmune phenotypes of both mice and man, i.e. the lack of anergic, suppressive T-Reg cells. These initial studies indicated that in the mouse, FOXP3 expression was restricted to CD4+CD25+ T cells, both in the thymus and in the periphery, thus consistent with the T-Reg phenotype. Moreover, *in vitro* stimulation of CD4+CD25- T cells by anti-CD3 together with anti-CD28 or IL2 failed to elicit FOXP3 mRNA expression. Accordingly, FOXP3 expression by CD4+CD25+ T cells was quickly adopted as a more definitive phenotypic definition of a T-Reg cell.

However, confounding these data were observations made with human T cells. Activation of human Peripheral Blood Mononuclear Cells (PBMC) via anti-CD3 was found to result in an increase of FOXP3 mRNA in CD4+ T cells [30]. Moreover, human CD8+ T cells were also found to express FOXP3 mRNA [31], [32]. These findings prompted the speculation that the expression of FOXP3 in an antigen-activated T cell could act as a natural negative feedback loop that would prevent unrestricted cytokine production and inflammatory reactions [31].

Recently, antibodies reactive with FOXP3 became available, facilitating experimental approaches in this field. Of interest, it was found that peak expression of FOXP3 in human T cells requires as long as three days after activation with anti-CD3 *in vitro,* thereby almost excluding an immediate/early FOXP3 gene activation via the TCR/CD3 complex [33]. Very recently, others reported that antibodies reactive with IL2 or the IL2 receptor diminished TCR/CD3-induced FOXP3 expression by CD8+ T cells [34]. By comparison, purified CD4+CD45+CD25-CD127+ human T cells activated with solid-phase anti-CD3+soluble anti-CD28 in the presence of both IL2 and TGFβ supplied exogenously results in FOXP3 expression by most remaining cells [35], [36]. In addition, other recent studies demonstrated that IL2 provides a negative feedback effect on its own production via Signal Transducer and Activator of Transcription-5 (STAT5), although the mechanism whereby this occurs was left unexplored [37].

Recently, using several experimental approaches CD4+CD25+FOXP3+ cells were shown to suppress effector CD4+ T cells not by affecting early TCR activation or proliferation of effector T cells. Instead, because CD4+CD25+FOXP3+ cells are anergic and cannot produce IL2, but can consume and degrade it, these cells induce cytokine deprivation-mediated apoptosis of effector T cells, both *in vitro* and *in vivo* [38]. Accordingly, we focused on the role of IL2 itself in the TCR/CD3-induction of FOXP3 expression by normal human T cells, as well as the effect of FOXP3 on IL2 expression by both FOXP3+ and FOXP3- cells. Our results, summarized in this report, are consistent with the interpretation that IL2-induced FOXP3 expression plays a larger role in immune regulation than has been conjectured until now, serving to provide for a negative feedback loop, restricting IL2 production. Thus, IL2 appears to have at least two important functions during T cell activation; 1) the promotion of proliferative clonal expansion of antigen-selected cells, and 2) a negative feedback mechanism that restricts IL2 production, thereby ensuring a self-limited proliferative response. By comparison, no evidence was found that FOXP3+ cells are capable of actively suppressing IL2 production by FOXP3- cells.

## Results

### FOXP3 expression by resting and anti-CD3-activated PBMCs

**Figure 1:**
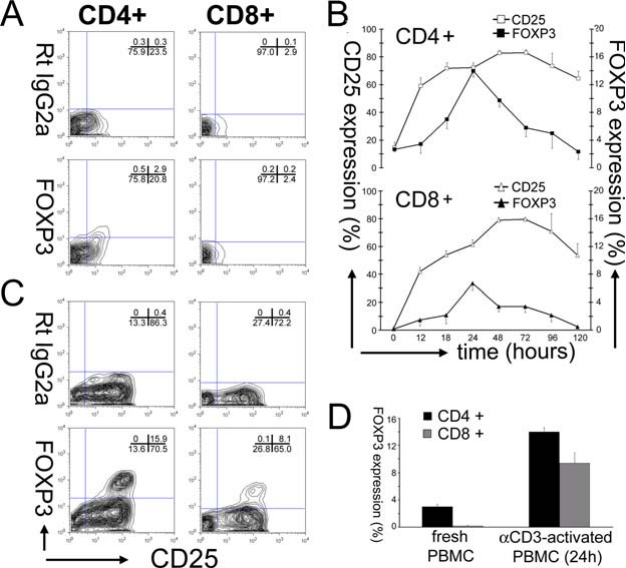
Differential FOXP3 expression by resting and anti-CD3-activated PBMCs. (A) Individual contour plots of freshly isolated total PBMCs representative of CD4+ (n = 19) and CD8+ cells (n = 19) stained with RatIgG2a (upper row) or FOXP3 (lower row) and IL2Rα (CD25). The percentages of the different cell populations are also shown for each quadrant of the contour plots in each upper right quadrant. (B) CD25 and FOXP3 expression in CD4+ cells (upper graph) and CD8+ (lower graph) cells were monitored daily upon activation of total PBMCs (t = 0). CD25 expression corresponds to the left axis (0–100%), while FOXP3 expression corresponds to the right axis (0–20%). The results are shown as Mean±SEM of 4 independent experiments. (C) Representative contour plots of total PBMC activated for 24 h show CD4+ and CD8+ cells stained with RatIgG2a (upper row) or FOXP3 (lower row) and CD25 as in A. The percentages of different population are also shown for the contour plots in each upper right quadrant. (D) The difference of FOXP3 expression between freshly isolated vs. αCD3-activated for 24 h in CD4+ (black bars; n = 16) and in CD8+ (gray bars; n = 17) cells. The results are shown as mean±SEM (vertical brackets).

A survey of 19 individuals revealed that only a few freshly isolated human T cells expressed detectable FOXP3, with a greater proportion in CD4+ T cells (2.64%±0.33%) (Mean±SEM, n = 19) vs. CD8+ T cells (0.16%±0.05%) (Mean±SEM, n = 19). The expression of FOXP3 vs. CD25 by freshly isolated CD4+ and CD8+ T cells is shown by representative contour plots in Figure 1A. It is noteworthy that only CD25+ cells express FOXP3, and that not all CD25+ cells are FOXP3+. Actually in the representative experiment shown (Figure 1A), only 12.2% of freshly isolated CD4+CD25+ T cells express FOXP3, and only 7.7% of freshly isolated CD8+CD25+ cells express FOXP3. It is also noteworthy that the level of both CD25 and FOXP3 expression is low as detected by the fluorescent intensities. According to standard terminology, the CD4+CD25+FOXP3+ cells would be “natural T-Regs”.

After activation with anti-CD3 the time course of FOXP3 expression by both CD4+ T cells and CD8+ T cells was analyzed by flow cytometry after successive intervals of culture (Fig 1B). Increased FOXP3 expression could first be detected above baseline after 12 hours, with peak levels observed at 24 hours in CD4+ T cells (13.97%±0.87 %) (Mean±SEM, n =  4) and CD8+ T cells 6.69±1.04% (Mean±SEM, n =  4). Thereafter, the frequency of FOXP3+ cells declined progressively, returning to baseline by 96 hours (day 4) of culture. For comparison, the expression of CD25 was also monitored (Fig 1B). It is readily apparent that CD25 expression precedes FOXP3 expression and eventually is detectable on >80% of both CD4+ and CD8+ T cells by 48 hours following activation, thereby attesting to the efficiency of anti-CD3 activation.

Representative 4-quadrant contour plots of T cells from PBMCs cultured with anti-CD3 for 24 hours are shown in Fig 1C, comparing FOXP3 expression and CD25 expression. In this typical example of 29 separate experiments, it is noteworthy again that only CD25+ cells express FOXP3, but not all CD25+ cells express FOXP3. Thus, even though 86.4% (15.9% +70.5%) of the CD4+ T cells became CD25+ after 24 hours, only 18.4% of the CD4+CD25+ cells also expressed FOXP3. Likewise, 73.1% (8.1%+65.0%) of the CD8+ became CD25+ after 24 hours, but only 11.1% of the CD8+CD25+ cells also expressed FOXP3. It is also important to notice that FOXP3 is only expressed by CD25+ cells that also express the highest levels of CD25, and that the level of FOXP3 expression is ∼10-fold higher than that of the freshly isolated cells, both CD4+ as well as CD8+, as detected by fluorescence intensity.

As shown in Fig 1D, the mean FOXP3 expression of freshly isolated T cells is compared with the mean FOXP3 expression after 24 hours of culture with anti-CD3. The FOXP3 expression of CD4+ T cells increased ∼5-fold from 2.99%±0.36% at baseline to 13.97%±0.61% (Mean±SEM, n = 16) after 24 hours culture (p = 0.0004), while the FOXP3 expression of CD8+ T cells increased ∼50-fold from 0.19%±0.06% at baseline to 9.38%±1.50% (Mean±SEM, n = 17) after 24 hours culture (p = 0.0003).

### Exogenous, recombinant IL2 prolongs FOXP3 expression

**Figure 2:**
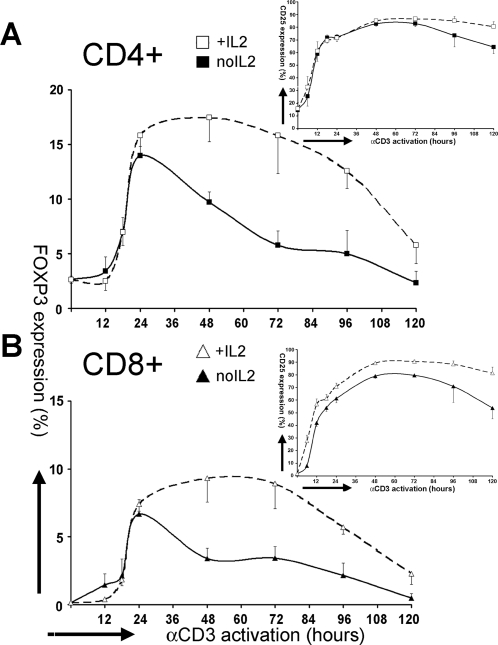
Exogenous, recombinant IL2 prolongs FOXP3 expression. The time course of FOXP3 expression in (A) CD4+cells and (B) CD8+ cells upon αCD3-activation of PBMCs (t = 0), with and without exogenous rIL2 [10 nM]. Insets show the IL2Rα (CD25) expression under the same conditions. The results are shown as Mean±SEM of 4–6 independent experiments.

As the time course of FOXP3 expression upon activation with anti-CD3 did not precede, but followed IL2R expression, we examined the effect of adding IL2 exogenously at t = 0 on the anti-CD3-induced FOXP3 expression. A saturating IL2 concentration (10 nM) did not accelerate FOXP3 expression by either CD4+ or CD8+ T cells (Fig 2). However, IL2 supplementation did prolong FOXP3 expression for 72–96 hours by both CD4+ T cells (Fig 2A) and by CD8+ T cells (Fig 2B), thereby suggesting that the duration of FOXP3 expression is dependent upon continuous signaling via the IL2/IL2R interaction. As shown in the insets, there was no change in the CD25 expression with the IL2 supplementation, which was already increased by 12 hours and became maximal by 48 hours after initiation of the cultures.

### Antibodies reactive with the both the IL2R and IL2 inhibit the anti-CD3-induced FOXP3 expression

**Figure 3:**
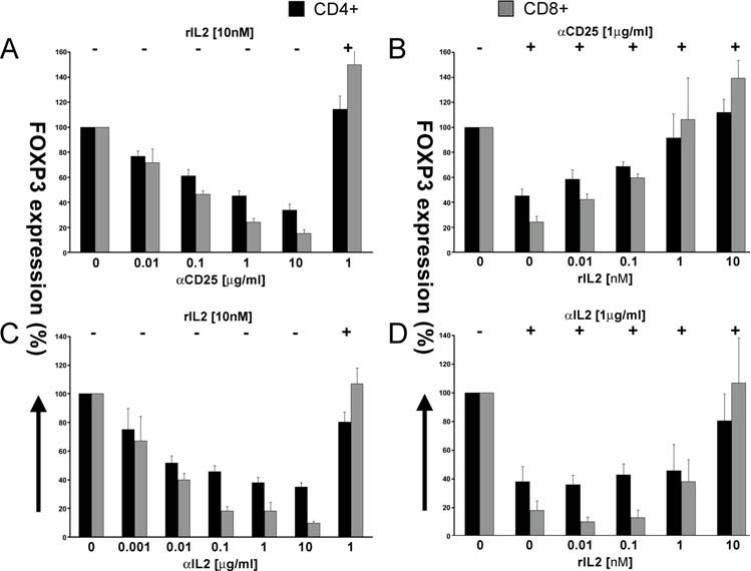
Antibodies reactive with the IL2Rα (CD25) and IL2 inhibit anti-CD3-induced FOXP3 expression. FOXP3 expression at 24 h in CD4+ (black bars) and CD8+ (gray bars) cells is shown upon αCD3-activation of PBMCs (t = 0), and after addition of increasing concentrations of (A) αCD25 and (C) αIL2 at t = 0. Exogenous rIL2 [10 nM] was added where indicated (t = 0). Also, the expression of FOXP3 at 24 h was determined in CD4+ (black bars) and CD8+ (gray bars) cells upon total PBMCs activation together with an addition of increasing concentrations of exogenous rIL2 [0–10 nM] at t = 0 in the presence of (B) αCD25 [1 µg/ml] and (D) αIL2 [1 µg/ml]. The results are shown as Mean±SEM of 4 independent experiments.

If the expression of FOXP3 induced by anti-CD3 triggering was actually secondary to anti-CD3-induced IL2 production and IL2R expression, followed by IL2/IL2R signaling, then antibodies reactive with the IL2R or with IL2 itself would be expected to inhibit FOXP3 expression. As shown in Fig 3A, anti-CD25 suppressed FOXP3 expression by both CD4+ and CD8+ T cells in a concentration-dependent fashion. Moreover, IL2 at an IL2R saturating concentration (10 nM) completely overcame the anti-CD25 suppression. That IL2 competed with the anti-CD25 is shown in Fig 3B, where increasing IL2 concentrations progressively circumvented the suppressive effect of a half-inhibitory concentration (IC_50 _∼1 µg/mL) of anti-CD25.

Confirmation of the IL2-dependent expression of FOXP3 upon TCR/CD3 triggering was obtained using antibodies reactive with IL2 itself. As shown in Fig 3C, increasing anti-IL2 concentrations suppressed anti-CD3-induced FOXP3 expression by both CD4+ and CD8+ T cells. Again, IL2 specificity was shown, in that this suppression could be competed by increasing IL2 concentrations (Fig 3D).

**Figure 4:**
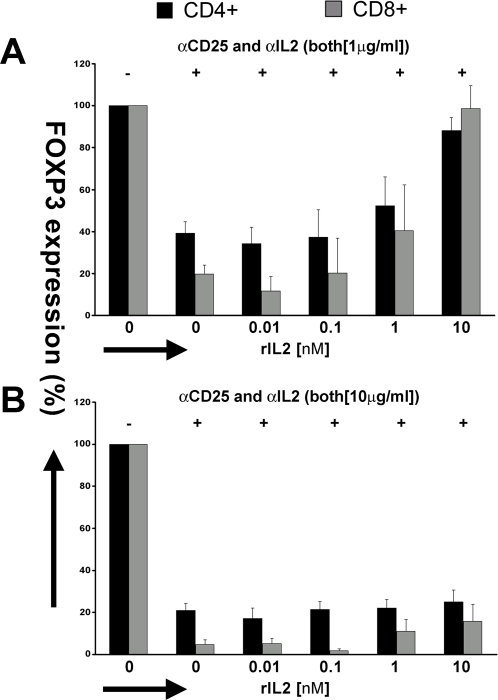
Inhibition of anti-CD3-induced FOXP3 expression by combined antibodies reactive with both IL2R (αCD25) and IL2. FOXP3 expression at 24 h is shown in CD4+ (black bars) and CD8+ (gray bars) cells upon αCD3-activation of PBMCs and simultaneous addition of αCD25 and αIL2 (t = 0) at two concentrations (A) [1 µg/ml] and (B) [10 µg/ml] along with increasing concentrations of rIL2 [0–10 nM]. The results are shown as Mean±SEM of 4 independent experiments.

Although antibodies that block either IL2 or the IL2R α-chain suppressed FOXP3 expression, neither antibody was completely inhibitory. Therefore, a combination of anti-CD25 and anti-IL2 was tested, at near saturating antibody concentrations as shown in Fig 4A. The combination of anti-IL2 and anti-IL2R α chain suppressed the anti-CD3-induced FOXP3 expression of CD4+ T cells by >60% and of CD8+ T cells by >80%, and exogenous IL2 supplementation was capable of overcoming the suppression mediated by the combined mAbs. However, as shown in Fig 4B, increasing the combined antibody concentrations 10-fold resulted in almost complete suppression of FOXP3 expression (80% for CD4+ and 95% for CD8+ T cells), which could not be overcome by excess IL2 (10 nM).

### Other γ_c_-chain cytokines have no effect on anti-CD3-induced FOXP3 expression

Although these experiments supported the interpretation that FOXP3 expression was regulated specifically by IL2, it was important to test other cytokines as well, especially those known to signal via the γ_c_-chain, which activates JAK3 and STAT5. As shown in Supplementary online material (Fig S1), there was no effect on anti-CD3-induced FOXP3 expression by mAbs reactive with IL4, IL7, IL15, or the IL21R.

### The expression of FOXP3 and IL2 are mutually exclusive

**Figure 5:**
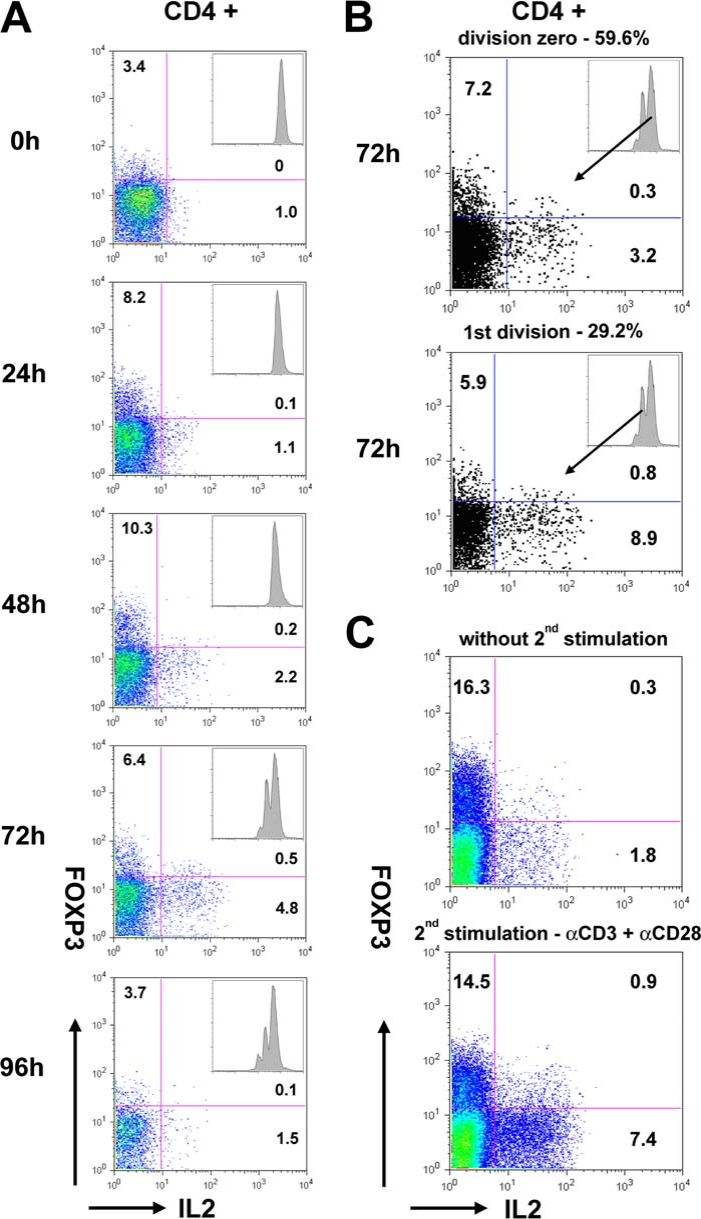
The expression of FOXP3 and IL2 are mutually exclusive. (A) Dot plots monitoring FOXP3 and IL2 expression in CD4+ cells measured on different days after αCD3-activation of PBMCs (t = 0). (B) FOXP3 and IL2 expression are shown for CD4+ cells in division zero (upper dot plot) and in the first division (lower dot plot) 72 h after total PBMCs activation (t = 0). Histograms of corresponding CFSE labeled CD4+ cells are shown in the insets. The percentages of the different populations are also shown for each dot plot. Representative dot plots were chosen from 1 of 4 independent experiments. (C) FOXP3 and IL2 expression are shown after restimulation. PBMCs were activated with anti-CD3+IL2 (10 nM) for 24 hours, harvested and then reactivated with anti-CD3+anti-CD28 in the presence of Brefeldin-A for 6 hours. Representative dot blots were chosen from 1 of 5 independent experiments.

Inasmuch as IL2 appeared responsible for stimulating the expression of FOXP3, and FOXP3 was reported to attenuate IL2 gene expression, the effect of FOXP3 on anti-CD3-induced IL2 production was examined. PBMCs were labeled with CFSE, and activated with anti-CD3. As shown in Figure 5A, CD4+ T cells were monitored daily for FOXP3 vs. IL2 expression, and for cell division by quantitative CFSE dilution (insets). At t = 0, all of the cells could be ascribed to a homogeneous single CFSE peak (Division = 0), and expressed low levels of FOXP3 (3.4%) and IL2 (1.0%). After 24 hours of culture, all of the cells remained undivided (Division = 0). At this time interval, none of the cells had divided, but FOXP3 had increased to 8.2%. Accordingly, the increased percentage of FOXP3+ cells could only have been derived from cells that were FOXP3- at t = 0. It is also noteworthy that few cells expressed both FOXP3 and IL2 (0.1%). After 48 hours of culture the separation of FOXP3+ (10.3%) vs. IL2+ (2.2%) cells was more evident and all of the cells still remained undivided. At this time, only 0.2% of CD4+ cells were doubly positive for FOXP3 and IL2 expression. This pattern persisted after 96 hours of culture, at which time cells had entered the second division. Moreover, as shown in Figure 5B, after 72 hours of culture when ∼30% of the cells had divided, the expression of FOXP3 and IL2 remained mutually exclusive, whether or not the cells had divided. Also, it was particularly noteworthy that the FOXP3+ cells did not “suppress” the expression of IL2 by FOXP3- cells.

### FOXP3 expression restricts but does not suppress IL2 expression upon TCR/CD3 restimulation

The observation that FOXP3 and IL2 expression were mutually exclusive is consistent with the hypothesis that FOXP3 functions to restrict IL2 expression by FOXP3+ cells, but does not actively suppress IL2 expression by FOXP3- cells. Accordingly, to test this hypothesis, PBMCs were activated for 24 hours with anti-CD3+IL2 (10 nM) to promote maximal FOXP3 expression. The cells were then harvested and reactivated for 6 hours with anti-CD3+anti-CD28, after which both FOXP3 and cytokine expression were monitored. In this experimental design, the cells are present in a physiologic setting, at relative concentrations that should allow detection of FOXP3+ cell active suppression of IL2 expression by FOXP3- cells. As shown by a representative experiment in Figure 5C, after TCR restimulation, 94.2% of the FOXP3+ cells remained IL2-. Moreover, the few detectable double positive cells (5.8%) expressed only low levels of FOXP3 as monitored by mean fluorescence intensity. By comparison, the presence of FOXP3+ cells did not actively suppress the IL2 expression by FOXP3- cells, which increased 4-fold from 1.8% to 7.4%. Thus, IL2 expression was restricted in FOXP3+ cells, but was not suppressed in FOXP3- cells.

Compilation of data from 5 individual experiments (Table S1, Supplementary online material) revealed that after restimulation, the mean IL2 expression by FOXP3- cells increased 10-fold, from 1.3%±0.4% (Mean±SEM) before restimulation to 12.6%±2.2% (Mean±SEM). Moreover, only 2.0%±0.4% (Mean±SEM) of cells expressed both FOXP3 and IL2. Accordingly, in this physiologic experimental setting, with a ratio of FOXP3+ to FOXP3- cells of 1∶5 (i.e. mean 16.5% FOXP3+ cells vs. 83.5% FOXP3- cells before reactivation), it was even more apparent that FOXP3+ expression restricted the expression of IL2, but FOXP3+ cells could not actively suppress the expression of IL2 by most FOXP3- cells.

## Discussion

These results support the conclusion that the IL2/IL2R interaction is responsible for most of the observed increased FOXP3 expression by TCR/CD3-activated human T cells. The specificities of anti-CD25 and anti-IL2, both of which compete solely for IL2-IL2R interactions, together with the lack of an inhibitory effect of mAbs reactive with other IL2Rγ_c_ cytokines, essentially leaves little possibility for any other interpretations. Moreover, finding that FOXP3 and IL2 expression are mutually exclusive, especially upon restimulation, leads inevitably to the interpretation that an IL2-FOXP3-mediated negative feedback loop regulates the antigen-activated T cell immune response by controlling IL2 production. However, we could find no evidence that FOXP3+ cells actively suppress IL2 production by FOXP3- cells.

Given these findings, it is inescapable that a deficiency in this negative feedback loop could underlie the hyperproliferative/autoimmune phenotypes of the IL2 (−/−) and *scurfy* mice, as well as in the XLAAD/IPEX patients. In this regard, there appears to be a FOXP3 gene dosage effect, in that males with an X-linked FOXP3 mutation and a complete lack of functional FOXP3 succumb to a rapidly fatal lymphoproliferative syndrome. By comparison, lack of IL2 as in the IL2 (−/−) mouse leads only to a diminution of FOXP3 gene expression, primarily in peripheral T cells, and results in a much slower accumulation of peripheral activated T cells and the lymphoproliferative/autoimmune syndrome [39]. Moreover, recently the gene region termed insulin dependent diabetes 3 (*Idd3)*, which promotes type 1 diabetes (T1D) development in the non-obese diabetic mouse, results in a 50% reduction of IL2 production, and this predisposes to T1D [40]. *Idd3* has also been associated with susceptibility to experimental autoimmune encephalomyelitis and autoimmune ovarian dysgenesis induced by neonatal thymectomy [40].

We purposely performed our *in vitro* stimulations solely with soluble anti-CD3 and total PBMC populations, thus relying on monocytes for presentation of the mAb and for costimulation, which most closely mimics physiological activation via the APC MHC-peptide antigen complex. In this regard, it is noteworthy that IL2/IL2R signaling of FOXP3 expression was so obvious, in that with the IL2/IL2R interaction blocked, the TCR/CD3-generated signals and any other cytokines produced *in situ*, such as TGFβ, could not increase the frequency of FOXP3+ cells. These observations are entirely consistent with those obtained utilizing either murine or human purified CD4+ T cells, and it is possible to selectively expand these cells with IL2 so that 100% become FOXP3+ after 5–7 days of culture [35], [36]. As the TCR/CD3 signaling complex is known to activate members of three families of transcription factors (i.e. Rel, AP-1 and NF-AT) [41], [42], while the IL2/IL2R activates STAT5 [43], our data are consistent with the interpretation that FOXP3 expression is regulated predominantly by STAT5 as has been shown recently by others [39], [44], [45].

Activation of murine T cells *in vitro* with anti-CD3+anti-CD28 was originally reported not to result in the expression of FOXP3 mRNA [27], [28], [29]. However, subsequently, it was found that TGF-β dramatically promotes the expression of FOXP3 mRNA by CD4+ T cells stimulated with anti-CD3/28, while TGF-β alone has no effect [46], [47]. More recently, IL2 was found to be essential for TGF-β-facilitated induction of anti-CD3 activation of murine T cell FOXP3 expression[47]. Moreover, other cytokines (IL-4, 6, 7, 9, 12, 15, 18, 21) failed to substitute for IL2. It has also been reported that murine CD4+CD25+FOXP3+ cells can be induced *in vivo* by antigen stimulation of naïve CD4+CD25- T cells [48], provided IL2 is available [49].

Accordingly, in both mice and humans, it appears that it is possible to convert FOXP3- T cells to FOXP3+ T cells by immunological activation. As well, in both species, this conversion requires IL2. In this regard, there have been several recent reports that focus on the mechanism(s) regulating FOXP3 gene expression. Mantel et. al. [50] explored the structure and function of the human FOXP3 promoter, and provided evidence that there is a proximal promoter localized between −511/+176 bp and that this region has at least 3 NF-AT response elements (RE), and 3 AP-1 REs, thereby implicating TCR regulation of FOXP3 expression. As well, others have identified a TCR-responsive enhancer in the first intron of the FOXP3 gene that is dependent upon a cyclic AMP response element binding protein/activating transcription factor site [51]. By comparison, Zorn and co-workers also examined regulation of the human FOXP3 gene, and provided evidence that IL2, via STAT5, promotes expression of FOXP3 by binding to a STAT RE located in the first intron of the FOXP3 gene [45]. Subsequently, Burchill and co-workers used the reductionist power of gene deletion experiments in the mouse to show convincingly that STAT5 is both necessary and sufficient for FOXP3 expression, and that there are 6 potential STAT5 REs in the gene, 3 in the promoter region and 3 in the first intron [39]. Moreover, using chromatin immunoprecipitation (ChIP) assays, evidence was presented that STAT5 binds to the FOXP3 gene promoter. Similar results have been reported recently by Yao and co-workers [44], who also show that STAT3 and STAT5a/b appear to have opposing roles in the regulation of FOXP3.

Given the expression of FOXP3 by TCR/IL2-activated T cells, what can be expected as to the outcome of the expression of this gene? Two functions have thus far been attributed to FOXP3, the restriction of TCR-signaled cytokine gene expression by FOXP3+ cells, and the active suppression of cytokine production by FOXP3- cells mediated by FOXP3+ cells. Our results demonstrate that FOXP3 and IL2 expression are mutually exclusive, consistent with the restriction of IL2 expression by FOXP3+ cells, but indicating that FOXP3+ cells do not actively suppress IL2 expression by FOXP3- cells, at least when both FOXP3+ and FOXP3- cells are present in a mixed population of PBMCs.

If CD4+CD25+FOXP3+ cells cannot actively suppress effector T cells, how can one account for the phenotypes of the *scurfy* mouse, and those individuals suffering from the XLAAD/IPEX syndrome? FOXP3 expression normally functions to down-regulate T cell activation, particularly cytokine gene expression. Actually, in the earliest studies of FOXP3 gene function, it was found that FOXP3 over-expression in CD4+ T cells attenuates activation-induced cytokine production and proliferation [52]. Moreover, several forkhead-specific REs adjacent to critical NF-AT REs in the promoters of several cytokine genes were identified.

Subsequently, the effect of FOXP3 expression vs. FOXP1 and FOXP2 was examined, and only FOXP3 specifically inhibited cytokine gene expression (IL2, IL4, IFN-γ) by primary murine CD4+ T cells. Furthermore, FOXP3 was shown to inhibit Rel family (NF-κB p65 & NFAT) transcriptional activation of cytokine genes without inhibiting their DNA binding activity [53]. In addition, they found that FOXP3 physically associates with NF-κB p65 and NFAT proteins and inhibits their capacity to transactivate. These results were confirmed and extended by others who showed that FOXP3 is capable of repressing CREB- as well as NF-κB-dependent transcription [54]. As well, the crystal structure of FOXP2 complexed with NF-AT was solved, revealing that this protein complex forms an extensive protein-protein interaction interface [55]. Moreover, mutations of FOXP3 predicted from this crystal structure to disrupt the FOXP2/NF-AT complex correlates with the loss of FOXP3-inhibition of IL2 gene expression.

Additional data have been recently reported on analyses of FOXP3 target genes by combining ChIP analysis with DNA arrays. Of interest, using these approaches, one group found that FOXP3 binds to ∼700 genes, and plays a dual role as both a transcriptional activator and repressor [56]. Another group used a FOXP3- murine T cell hybridoma transduced with FOXP3 and found IL2 gene expression to be suppressed only in FOXP3+ cells [57]. Thus, many FOXP3-regulated genes that encode proteins associated with the TCR signaling pathway have been identified, most of which showed suppressed activation when the cells were TCR-stimulated.

By comparison with the wealth of these data, there have been only two recent reports regarding possible molecular mechanism(s) responsible for the putative T-Reg active suppression of IL2 production and resultant proliferation, both of which have invoked an increase in intracellular cAMP [58], [59]. To date, the *in vitro* T cell proliferation assays for the capacity of T-Reg cells to suppress effector T cells have utilized high ratios of T-Regs to effectors, a situation that favors the inhibition of proliferation by the consumption of the T cell growth factor, IL2. In as much as T cell proliferation is dependent upon the concentration of IL2 available as demonstrated in our earliest studies [1], because FOXP3+ cells are restricted in their capacity to produce IL2, but can consume any IL2 produced by effector cells, decreased IL2-driven T cell proliferation in the presence of high ratios of FOXP3+ : FOXP3- cells (e.g. 2∶1 to 1∶4) would necessarily be more apparent than real. Thus, aided by new assays to detect the accumulation of intracellular IL2, rather than its concentration in the surrounding media or its growth promoting effects, we could detect no evidence that FOXP3+ cells can actively suppress IL2 expression by FOXP3- cells.

It is worthy of emphasis that these findings are entirely consistent with the recent report of Pandiyan and co-workers [38], who showed that CD4+CD25+FOXP3+ cells did not suppress effector T cell production of IL2 or proliferation early after activation by anti-CD3 and anti-CD28, with or without APCs, thereby indicating that APCs are not essential for T-Reg cell-mediated suppression. Moreover, their data indicate that CD4+CD25+FOXP3+ cells consume IL2, which leads inevitably to cytokine deprivation-induced apoptosis of effector T cells, both *in vitro* and *in vivo*.

Thus, our findings are entirely consistent with the capacity of FOXP3+ T cells to *passively* suppress ongoing proliferative immune responses by reducing the concentrations of IL2 available to effector cells, both *in vitro* and *in vivo*. When the IL2 concentration is limiting, IL2R+ cells rapidly cease proliferating, and undergo cytokine-withdrawal apoptosis [38]. *In vivo* this mechanism is responsible for the retraction of >90% of T cells that expand after an initial antigen stimulation [60], [61]. This same mechanism could explain why attenuation of FOXP3 expression by genetic manipulation eradicates the suppressive function but not the restrictive function of FOXP3 both *in vitro* and *in vivo*, in that the FOXP3-attenuated cells only express low levels of IL2 receptors [62].

From all of these various experimental approaches, the emerging picture is that FOXP3 expression in peripheral mature T cells both in mice as well as humans is dependent upon TCR/CD28 triggering of IL2. Moreover, the most consistent consequence of IL2-induced FOXP3 expression is the restriction of IL2 gene expression in response to TCR activation as well as reactivation. Therefore, all of these data indicate that FOXP3 plays a key role in the feedback down-regulation of T cell activation and proliferation via the regulation of IL2 gene expression, thereby functioning normally to dampen immune responses. This interpretation is entirely consistent with the hyperproliferative/autoimmune phenotypes of the IL2 (−/−) and *scurfy* mice as well as XLAAD/IPEX patients.

However, no evidence could be found for an active suppressive effect of FOXP3+ cells on FOXP3- cell IL2 expression. Our data thus question the relevance of FOXP3 as an exclusive marker for T-Regs, as has been emphasized recently by others [63], as well as the role of FOXP3 in T-Reg-mediated *active* suppression. Accordingly, further experiments will be required to clarify the postulated active vs. passive suppressive roles in regulating IL2 availability, as compared with the demonstrable restrictive role of FOXP3 in regulating IL2 expression. As well, whether other mechanisms are responsible for active suppression of effector cell proliferation remains to be investigated. However, it is clear that the IL2 regulation of FOXP3 and the FOXP3 regulation of IL2 will now assume a central role in many diseases of the immune system.

Since the point of control over immune reactivity occurs at IL2 gene expression, it is logical that defects in the IL2/FOXP3 negative feedback loop may lead to a preponderance of activating signals and autoimmunity. Conversely, if the pivotal point of IL2 gene regulation by FOXP3 is circumvented, for example by autonomous signaling via persistently active JAK3/STAT5, then malignancy could result, as shown experimentally by over expression of STAT5 [64].

The therapeutic implications of our findings are obvious. In those situations where it can be documented that there is a deficiency in the IL2/FOXP3 negative feedback loop, such as in NOD mice, IL2 therapy may be beneficial. Moreover, blockade of the negative feedback loop could be used to enhance immune responses, for example for persistent infections, as anti-cancer therapy, and to enhance the efficacy of vaccines. Finally, FOXP3+ T cells could be used as adoptive therapy to dampen unwanted immune responses, not by actively suppressing activated effector cells, but by passively removing IL2.

## Materials and Methods

### Peripheral Blood Mononuclear Cell (PBMC) Isolation, Culture and Activation

PBMCs from normal volunteers were isolated from whole blood using centrifugation over a Ficoll-Hypaque density gradient. Total PBMCs were cultured in 12-well plates (BDLabware) (3×10^6 ^cells/mL), and were activated with anti-CD3 (OKT3, Ortho Biotech, 1∶800,000 dilution) in complete cell culture media (RPMI 1640 with L-glutamine (Cellgro), supplemented with 10% FBS, 100 U/mL Penicillin/Streptomycin (both Gemini Bio-Products) and 2 µM 2-ME (Sigma-Aldrich). The following antibodies were added simultaneously, when indicated: anti-CD25, anti-IL2, anti-IL4, anti-IL9, anti-IL15 and anti-IL21R antibodies (all R&D Systems) and human rIL-2 (Amgen). All cells were cultured in a 37°C humidified incubator with 5% CO_2_.

### Proliferation and Cytokine Production

In some experiments, freshly isolated PBMCs were first labeled with 5 µM 5- (and 6-) carboxyfluorescein diacetate succinimidyl ester (CFSE) (Molecular Probes) prior to culture. PBMCs were then activated (anti-CD3) and cultured for 96 h. For analysis of intracellular cytokine production in cultured, CFSE-labeled and activated PBMCs, Brefeldin A (10 µg mL^−1^, Sigma) was added for the final 6–18 h prior to cytokine staining daily for at least 4 days after activation. Anti-CD28 (10 µg mL^−1^, BD Biosciences) was used for co-stimulation where indicated.

### Monoclonal Antibodies (mAbs)

The following mAbs were used all reactive with human molecules: surface mAbs ; Ms IgG_1_,κ FITC, CD25 FITC, CD4 PerCp, CD8 PerCp, CD8 APC; intracellular mAb; IL2 PE (BD Biosciences); intranuclear antibodies reactive with FOXP3 (clones PCH101 and 236A/E7 from eBioscience; clone 259D from BioLegend) PE or APC, Rat Isotype IgG2a control (eBiocsience), Ms IgG1, κ (BioLegend). The specificity of the monoclonal antibody reactivity with FOXP3 was confirmed using FOXP3-transfected 293 cells kindly provided by Steven Jacobson (NINDS). eBioscience mAbs (clones PCH101 and 236A/E7), Biolegend clone 259D, and Abcam polyclonal antibodies (ab4728), showed similar patterns and specific reactivity with FOXP3 using FOXP3-transfected 293 cells and human PBMCs.

### Flow Cytometry

Four-color flow cytometry and the eBioscience PE and APC FOXP3 staining sets with modified eBioscience staining protocols were used. Single cell suspensions of lymphocytes were stained first for surface molecules for 30 minutes in the dark (room temperature), then washed once with Washing Buffer (1X PBS (Cellgro) with 0.5% FBS (Gemini Bio-Products) and 0.1% NaN3). The cell pellet was resuspended and 0.5 ml of freshly prepared eBioscience Fixation/Permeabilization Buffer was added to each sample and samples were incubated 45 minutes in the dark (4°C). The cells were washed once with Washing Buffer and then washed again by adding 1X Permeabilization Buffer; anti-human FOXP3 or Isotype Rat IgG2a control antibody (and intracellular antibodies when stained) was added and samples were incubated for 30 minutes in the dark (room temperature). The cells were then washed with Washing Buffer, acquired on a FACSCalibur flow cytometer, and the data were analyzed using FlowJo software (Tree Star) using 2% Contour plots to set quadrants.

### Statistical Analysis

Nonparametric Wilcoxon matched-pairs signed-rank test was performed in the analysis of mean differences in FOXP3 expression between freshly isolated and anti-CD3-activated CD4+ and CD8+ T subsets for 24 h. *P* values of <0.05 were considered statistically significant.

## Supporting Information

Figure S1 Other γc-chain cytokines have no effect on anti-CD3-induced FOXP3 expression. FOXP3 expression at 24 h is shown in CD4+ (black bars) and CD8+ (gray bars) cells upon αCD3-activation of PBMCs (t = 0) and simultaneous addition of (A) αIL4 and αIL15 or (B) αIL7 and αIL21R at concentrations of [1 µg/ml] and [10 µg/ml] along with rIL2 [10 nM] where indicated. Data shown represent the mean±SEM of 4 separate experiments.
(2.86 MB TIF)

Table S1 Reactivation of PBMCs with anti-CD3+anti-CD28, followed by monitoring for FOXP3 and IL2 expression. PBMCs were activated with anti-CD3+IL2 (10 nM) for 24 hours, then harvested and cultured without or with restimulation by anti-CD3+anti-CD28 for 6 hours in the presence of Brefeldin-A. The percentage of CD4+ T cells positive for FOXP3, IL2 and both FOXP3+IL2 are listed from 5 individuals, together with the mean±SEM.
(0.02 MB DOC)
