# Supplementary figures and images for: Negative Feedback Regulation of T Cells via Interleukin-2 and FOXP3 Reciprocity
Source: PLoS One. 2008 Feb 13;3(2):e1581. doi: 10.1371/journal.pone.0001581 (PMC2265256; doi:10.1371/journal.pone.0001581)

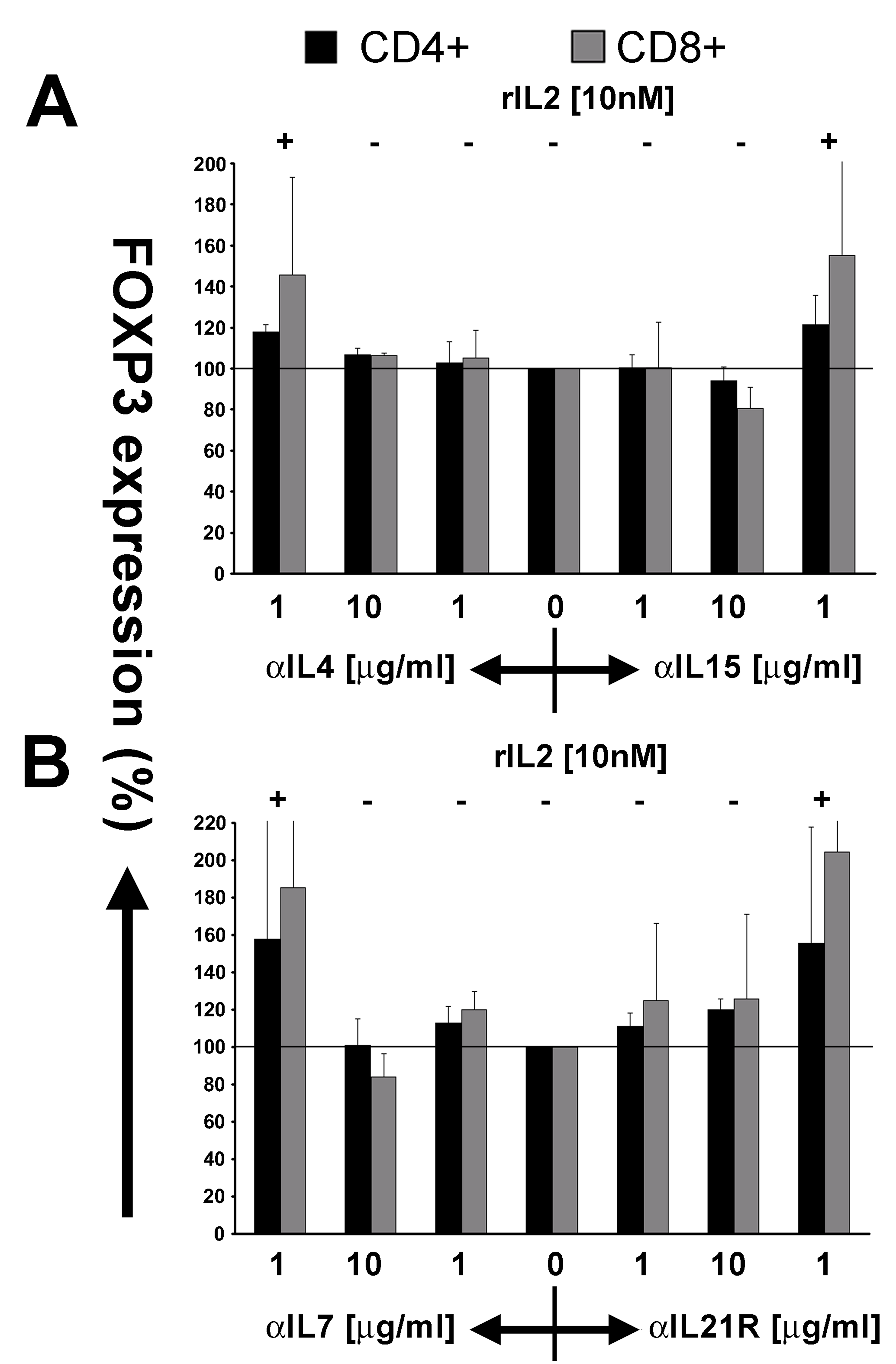

Supplement: Figure S1 — Other γc-chain cytokines have no effect on anti-CD3-induced FOXP3 expression. FOXP3 expression at 24 h is shown in CD4+ (black bars) and CD8+ (gray bars) cells upon αCD3-activation of PBMCs (t = 0) and simultaneous addition of (A) αIL4 and αIL15 or (B) αIL7 and αIL21R at concentrations of [1 µg/ml] and [10 µg/ml] along with rIL2 [10 nM] where indicated. Data shown represent the mean±SEM of 4 separate experiments. (2.86 MB TIF) [file pone.0001581.s001.tif]
