# Supplementary material for: Negative Feedback Regulation of T Cells via Interleukin-2 and FOXP3 Reciprocity
Source: PLoS One. 2008 Feb 13;3(2):e1581. doi: 10.1371/journal.pone.0001581 (PMC2265256; doi:10.1371/journal.pone.0001581)
Supplement: Table S1 — Reactivation of PBMCs with anti-CD3+anti-CD28, followed by monitoring for FOXP3 and IL2 expression. PBMCs were activated with anti-CD3+IL2 (10 nM) for 24 hours, then harvested and cultured without or with restimulation by anti-CD3+anti-CD28 for 6 hours in the presence of Brefeldin-A. The percentage of CD4+ T cells positive for FOXP3, IL2 and both FOXP3+IL2 are listed from 5 individuals, together with the mean±SEM. (0.02 MB DOC) [file pone.0001581.s002.doc]

Supplemental Table 1. Expression of FOXP3 and IL2 upon TCR stimulation
